# Alien Plants of Kyrgyzstan: The First Complete Inventory, Distributions and Main Patterns

**DOI:** 10.3390/plants13020286

**Published:** 2024-01-18

**Authors:** Alexander N. Sennikov, Georgy A. Lazkov

**Affiliations:** 1 Botanical Museum, Finnish Museum of Natural History, University of Helsinki, 00014 Helsinki, Finland; 2 Institute of Biology, Academy of Sciences of Kyrgyzstan, Bishkek 720010, Kyrgyzstan; glazkov1963@mail.ru; 3 Research Centre for Ecology and Environment of Central Asia, Bishkek 720040, Kyrgyzstan

**Keywords:** Asteraceae, Central Asia, checklist, cultivated plants, distribution, inventory, naturalisation, plant invasions, vascular plants, weeds

## Abstract

The first inventory of casual and naturalised alien plants of Kyrgyzstan is based on an overview of published data, which were re-assessed and re-evaluated using modern standards. Altogether, 151 alien species were registered in the country, of which nearly 40% became naturalised. The total number of alien plant species and the proportion of casual aliens are relatively low due to the harsh climatic conditions (high aridity and continentality) and predominantly high elevations. The highest number of alien plant species in Kyrgyzstan originated from the Mediterranean, which can be explained by some common climatic features between this area and Central Asia, but half of the ten most harmful aliens originated from the Americas. The intensity of plant invasions was the greatest during the period of the Russian Empire and the USSR, and this rapid accumulation of alien plants continues in independent Kyrgyzstan. The uneven distribution of alien plants in Kyrgyzstan is explained by different elevations and climatic conditions across its regions, as well as by the concentration of agricultural activities and human population along warm lowland depressions. More research is required to uncover pathways and particular times of introduction and to produce detailed distribution maps.

## 1. Introduction

Alien, or non-native vascular plants, have a variety of detrimental effects [1,2]. They have a deleterious impact on native ecosystems [3], produce a considerable economic impact on agriculture and industry [4], including infrastructure [5], and affect human health and well-being [6,7]. They outcompete native plants by physical competition [3] or allelopathic influence [8], and can threaten the gene pool of native plants by interspecific hybridisation [9]. Moreover, their negative impact on native ecosystems may also affect native fauna [10]. Alien plants may lessen the potential of protected areas to sustain the diversity of endangered plants [11]. Economic and environmental losses caused by alien plants require urgent legal actions from governments and other policy makers [12]. To cope with the problem, plant invasions should also be addressed at an international level [13].

To be capable of producing a detrimental effect, a plant species must first go beyond human control. This could be, e.g., unwanted contaminants of food and other commodities, which may subsequently become noxious weeds or ruderal plants, or intentional introductions, such as originally being used as a commodity but unintentionally escaped [14,15]. For example, ornamental plants may be more likely to escape from cultivation and become invasive, and their role in polluting native ecosystems is growing steadily [16]. In general, plant invasions of any origin result in significant decreases in the abundance and diversity of native plant species in natural biosystems; however, the productivity of such biosystems increases. This observation shows that invasive plants may effectively replace native plant diversity in terms of biomass and ecosystem function [17].

Recent decades have seen great progress in inventories of non-native plants, which covered several national and regional territories [18]. These inventories contribute to the Global Naturalized Alien Flora database, which includes lists of alien plant species in 1029 regions with residence status provided for each species record [19]. Nevertheless, countries of Central Asia have not been involved in active studies of alien vascular plants. Thus, no national lists of alien plants have been produced in Kazakhstan, Tajikistan or Turkmenistan.

In Uzbekistan, a list of alien plants [20] has been recently compiled for the Global Register of Introduced and Invasive Species [21]. A systematic inventory of alien plants is on-going within the ‘Flora of Uzbekistan’ Project [22], with detailed information on species distribution and residence status published in every volume of the Flora [23,24,25,26,27,28]. Important new records may be detailed separately on case-by-case basis (e.g., [29]).

In Kyrgyzstan, a similar list of alien plants exists [30]. It was largely based on the vascular plant checklist of Kyrgyzstan [31], to which assessments of residence status (native vs. alien) were added on the basis of the previously published floristic information [32,33,34,35,36,37,38,39,40,41,42,43,44,45,46] and the author’s field observations. Several smaller contributions included further new records of vascular plants [47,48,49,50,51]. Finally, a checklist of vascular plants in the Tian-Shan mentioned the residence status of all species recorded in this mountain system [52], but it did not cover the southern part of Kyrgyzstan which belongs to the Pamir-Alay Mountain System. A detailed overview of studies of alien plants in Kyrgyzstan can be found in our previous work [53].

A monographic revision of alien vascular plants of Kyrgyzstan has been started recently [53,54]. The purpose of this on-going project is to collect every historical record of alien vascular plants in Kyrgyzstan, with their evaluation in the context of historical events and plant introductions in Central Asia as a whole. During this work, identifications are revised in complicated taxonomic groups. The revision is accompanied by point maps documented by herbarium specimens or photographs. The pathways and timing of introduction are determined or inferred for each record, and the resulting data are matched against historical processes obtained from the pattern of plant invasions in other countries of Central Asia.

Despite the rich data already uncovered by Sennikov & Lazkov [53,54], this work progresses slowly and currently provides detailed information on a fraction of alien plants of Kyrgyzstan. Furthermore, a brief format and the static nature of the previously published checklist [30] does not allow for the information on alien plants of Kyrgyzstan to be used in broad-scale analyses of global tendencies in plant invasions. For this reason, in the present work, we provide an updated list of non-native vascular plants of Kyrgyzstan with their attributes, as much detailed as possible but based on the currently available information.

As long as the database of historical records of alien vascular plants in Kyrgyzstan [55] remains incomplete, we are unable to uncover detailed distribution patterns, introduction periods and pathways for the whole list of alien plants of the country. Nevertheless, we can provide draft assessments of the residence status and invasion activity of every species, together with their estimated current impact on native environments, agriculture and human well-being, with distribution schemes based on the phytogeographic districts developed by Sennikov & Lazkov [53]. The published assessments [53,54] provide instructive examples of vectors and pathways of introduction, which reflect important trends in the history of plant invasions.

The present contribution adds to the current progress in botanical studies of Central Asia, in which updates of the existing information and data mobilisation belong to the most urgent tasks for the future [56]. The first complete list of alien plants of Kyrgyzstan presented here aims to provide the basis and starting point for the future detailed revision and analysis of plant invasions in Kyrgyzstan, which are required in order to develop informed actions in nature protection, agriculture and urban planning.

## 2. Materials and Methods

### 2.1. Study Area

Kyrgyzstan is a landlocked and almost completely mountainous country in Central Asia. Its territory totals 199,951 km^2^. The greatest part of the country is occupied with high mountains and their foothills, with the Tian-Shan (to the north-west, north and east) and Pamir-Alay (to the south-west) being the leading mountain systems. The Fergana Depression and the Chüy Valley, the largest fertile lowlands surrounding the country, are only marginally owned by Kyrgyzstan. The territory lies between a latitude of 39°78′ and 42°07′ north and a longitude of 72°24′ and 74°19′ east, stretching for nearly 1000 km from west to east and 450 km from north to south. Altitudes vary between 395 and 7440 m but largely remain above 1500 m, with a minor (5%) proportion of lowlands. The climate is predominantly arid and highly continental [57,58].

Kyrgyzstan shares borders with Kazakhstan, Uzbekistan, Tajikistan and China. Its geographic location defines a large part of its economic connections, whereas the other part is explained by its common history with the former Soviet Union. Agriculture is largely confined to the warm depressions of the northern and western parts of the country, where fields and orchards are situated, although the smaller-scale cultivation of crops and foraging also occur in the mountains. Mining and its infrastructure development is greatly widespread, thus accounting for extensive anthropogenic disturbance in remote mountainous regions. Kyrgyzstan is a developing country with an actively growing population, which is predominantly rural.

**Figure 1:**
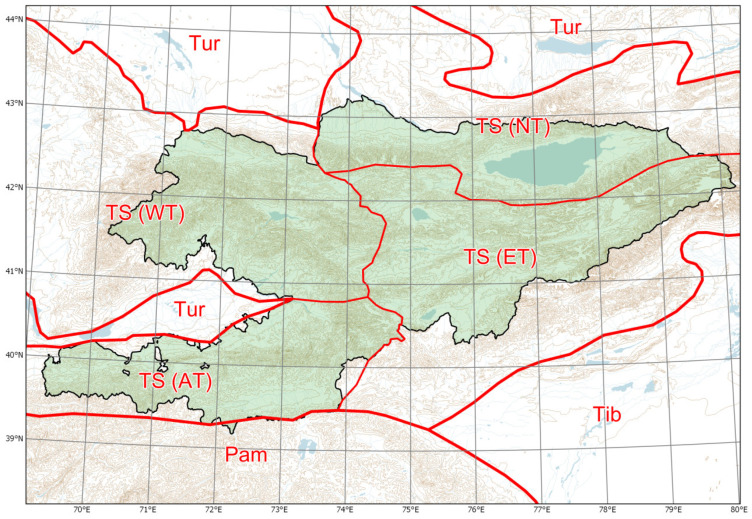
Major phytogeographic regions of Kyrgyzstan. Divisions (thick lines): TS (Tian-Shan), Tib (Tibet), Tur (Turanian), Pam (Pamir). Subdivisions (thin lines): AT (Alay-Turkestan), ET (Eastern Tian-Shan), NT (Northern Tian-Shan), WT (Western Tian-Shan). Source: [53], published and distributed under the Creative Commons Attribution License (CC BY 4.0).

Botanically, the territory of Kyrgyzstan is subdivided ([53]; Figure 1) into three main divisions: Turanian (highly arid lowlands), the Tian-Shan (more humid mountain system with greater plant diversity, including the Alay and Turkestan Ranges) and the Pamir (high and arid mountains with lower plant diversity). The Tian-Shan is further subdivided according to major distribution patterns and centres of endemism [52].

Altogether, 3927 species were listed as native or alien to Kyrgyzstan in the latest checklist [31], with additions from recent floristic records [50,51,53,54,59,60] and new species descriptions [61,62], although some species were recently removed from the list due to clarifications of synonymy or attribution [52,54,63,64]. We expect that the actual vascular plant diversity may reach 4000 species in the future because of the constant flow of new field records, on-going synanthropisation and taxonomic research on apomictic plants [64,65,66,67].

### 2.2. Data Collection

A list of alien plants of Kyrgyzstan is ultimately based on the floristic checklist [31], which is updated from numerous floristic and taxonomic publications on a regular basis. Taxonomy and nomenclature are updated after [52]. The family-level classification follows APG IV [68]. Published occurrences are supported by references to the latest synoptic work [31] or its supplements [50,51] and revisions [53,54].

Classical floristic inventories in Kyrgyzstan rarely distinguished between native and alien plants, except for neophytes that escaped from cultivation or arrived by unintentional means. Old neophytes and archaeophytes therefore went unrecognised [53]. In the present paper, the alien status for old introduced plants was largely borrowed from our previous works [30,52,53,54] with some corrections.

Neophytes were recorded as occurring spontaneously when they were located far outside the place of their original intentional introduction. Occasional seed set or garden waste occurring within cultivated land were not considered as long as land management was continuous.

We compiled the following characteristics for each alien species: life cycle, habitat type and native distribution area. We also recorded weedy behaviour (occurrence as a weed in fields or gardens), presence in cultivation (as ornamental, edible, technical/forage or medicinal), presence in native landscapes, and presence in ruderal and roadside situations. We estimated the time of introduction based on the following historical periods: pre-historical (or neolithic; ancient crops and their weeds), Islamic (starting from the Arabic invasion that brought many cultivated plants and intensified trade and cultural connections), Russian (or Soviet; trade with and transport from Eastern Europe), and independence (recent trade and cultivation). Moreover, we assessed residence status (casual vs. established) and applied IUCN EICAT categories and criteria [69] for the invasion status. Literature data [32,33,34,35,36,37,38,39,40,41,42,43,44,45,46] and personal field knowledge were used in the assessments.

The resulting checklist was published through the Global Biodiversity Information Facility [70]. Besides the checklist, this dataset contains distributional data collected according to phytogeographic regions [53].

Due to the lack of detailed inventories of distributional records, we were unable to accurately and reliably assess pathways [71] and more precise times of introduction (first records denoting certain pathways), or to provide a detailed classification of plant habitats in the field. These details are a subject of our separate on-going study [53,54].

### 2.3. Data Analysis

Proportional statistics were calculated to understand the composition and structure of the diversity of alien plants in Kyrgyzstan. The resulting information was evaluated against major patterns in human history, climate and plant geography.

## 3. Results

According to our literature survey, 151 species of vascular plants reported from Kyrgyzstan can be considered alien in the country.

**Figure 2:**
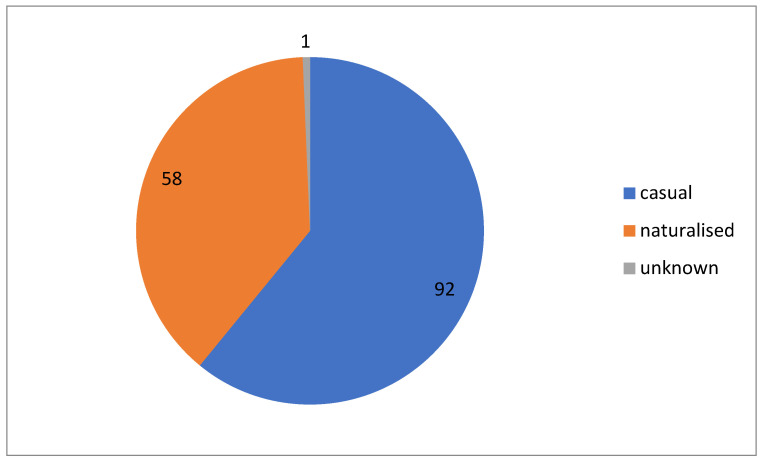
Residence status of alien plants in Kyrgyzstan, shown in the number of species.

By residence status (Figure 2), 58 species were classified as naturalised, 92 species as casual, and 1 case was considered uncertain. The level of naturalisation is 38.4%.

**Figure 3:**
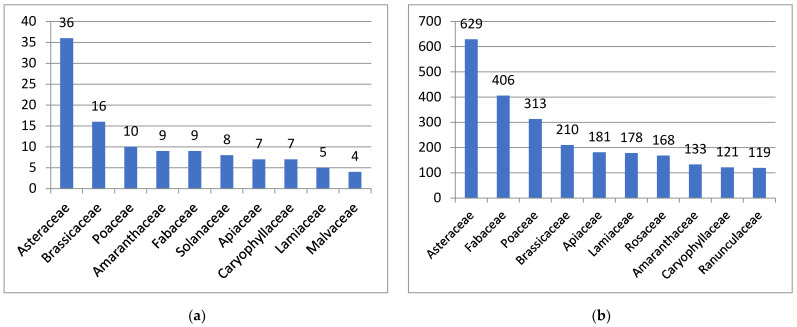
Top ten leading families of alien (**a**) and native (**b**) plants in Kyrgyzstan, shown in the number of species.

The alien species comprise 38 families, of which Asteraceae (36 species) is unambiguously leading. Brassicaceae (16 species) is the second, whereas a large suite of further families follows, starting with Poaceae (10 species). The top leading families (Figure 3a) are typical of arid zones, similar to those recorded in Iran [72].

The top ten native plant families (according to [31], with additions and corrections) is considerably different (Figure 3b), with a lower position of Brassicaceae and Amaranthaceae, and a higher proportion of Fabaceae, Rosaceae and Ranunculaceae. The latter two families surprisingly have a very low number of aliens in Kyrgyzstan.

**Figure 4:**
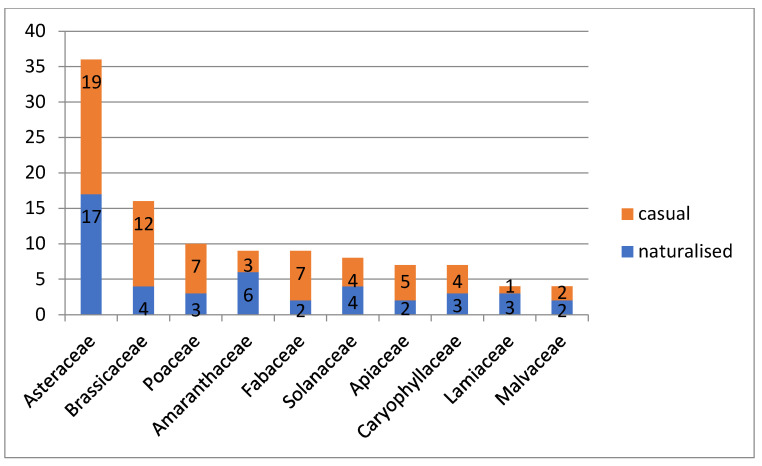
Proportion of casual and naturalised species in top ten leading families of alien plants in Kyrgyzstan.

The families Asteraceae, Amaranthaceae, Solanaceae and Brassicaceae include the greatest number of naturalised species, whereas Asteraceae, Brassicaceae, Fabaceae, Poaceae and Apiaceae are characterised by the greatest number of casual aliens (Figure 4).

The number of species per genus is very low due to a large number (119) of genera involved. Only six genera have a number of species greater than two: *Amaranthus* (6), *Vicia* (5), *Erigeron*, *Lathyrus*, *Sonchus* and *Xanthium* (3).

The alien species are almost exclusively terrestrial, with a single exception of the aquatic *Vallisneria spiralis*.

**Figure 5:**
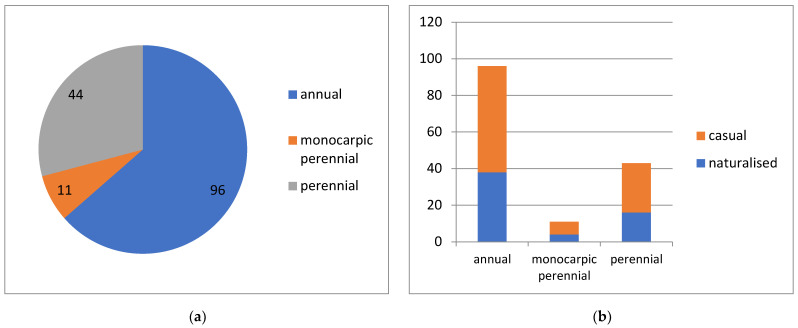
Classification of alien plants in Kyrgyzstan according to their life cycle (**a**) with their success of naturalisation (**b**).

Regarding life cycles (Figure 5), the greatest majority of alien species are annual or biennial, and this figure can be further increased with the addition of perennial monocarpic plants. Polycarpic plants constitute only 29% of the total alien flora. The success of naturalisation is similar in all three of these groups.

**Figure 6:**
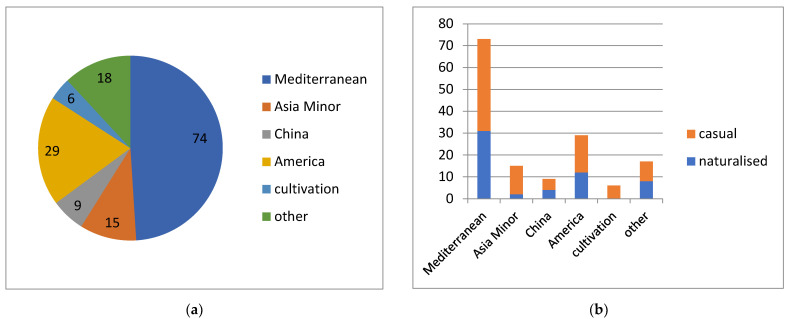
Native distribution areas of alien plants in Kyrgyzstan, classified according to their geographic types (**a**), with their success of naturalisation (**b**).

For the greatest majority of alien species, we were able to associate their native distribution areas with certain continents or their major regions (Figure 6a). We found that nearly half of the alien plant diversity originated in the Mediterranean area (occurring in the Mediterranean Basin and possibly stretching into the Caucasus and Iran), with the addition of a further 10% associated with its eastern areas only (Asia Minor to Iran). Nearly 20% originated from the New World. Minor yet considerable additions originated from China (6%) and from cultivation (4%).

The success of naturalisation (Figure 6b) is even nearly at 50% in most of these groups except in cultigenous plants (no naturalisation) and the East Mediterranean species (low level of naturalisation).

The majority of alien plants (93%) originated from areas with a temperate climate, whereas tropical areas provided only a minor fraction (7%) of the total species. Tropical species have shown no naturalisation success in Kyrgyzstan.

**Figure 7:**
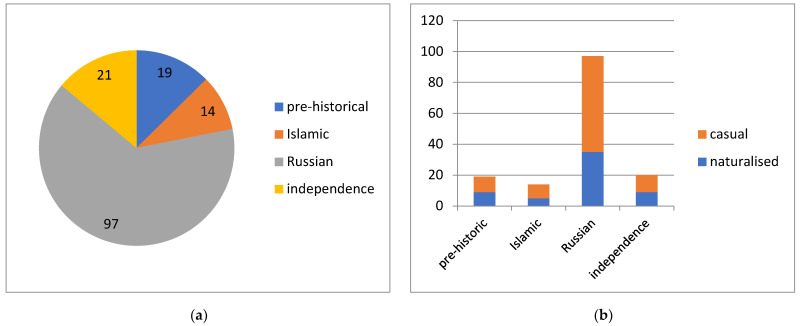
Major introduction periods of alien plants in Kyrgyzstan (**a**) with their success of naturalisation (**b**).

By the period of introduction, 9–13% of alien plants were introduced during each of the Neolithic, Islamic and independence periods, whereas nearly 65% arrived during the period of the Russian rule (Figure 7a). The level of naturalisation was at 43–47% among the plants that arrived during the Neolithic and independence periods, and among those that arrived during the Islamic and Russian periods, 35–36% of alien plant species became naturalised (Figure 7b).

**Figure 8:**
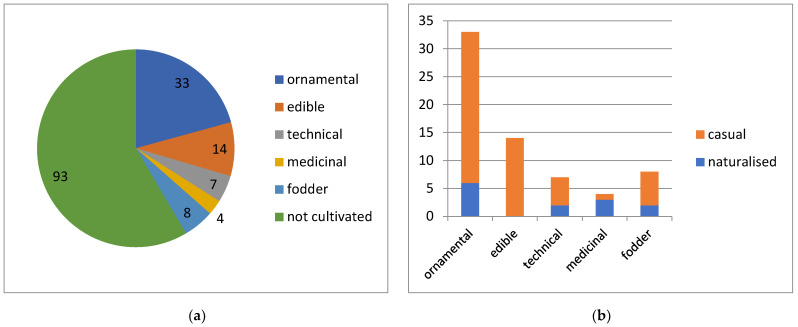
Cultivation purposes of alien plants in Kyrgyzstan (**a**), with their success of naturalisation (**b**).

Approximately 38% of alien plants were cultivated at some period in their history (Figure 8a). Of these, 21% were cultivated as ornamental plants, 9% as edible plants, 4.5% were cultivated as technical plants and the same number as fodder, and 3% were cultivated as medicinal plants; over 60% were not cultivated.

The success of naturalisation of these cultivated plants (Figure 8b) was different: a minor proportion (15–30%) of ornamental, technical or fodder plants was naturalised, whereas nearly all medicinal plants are established, and no edible plants can be considered naturalised in the country.

**Figure 9:**
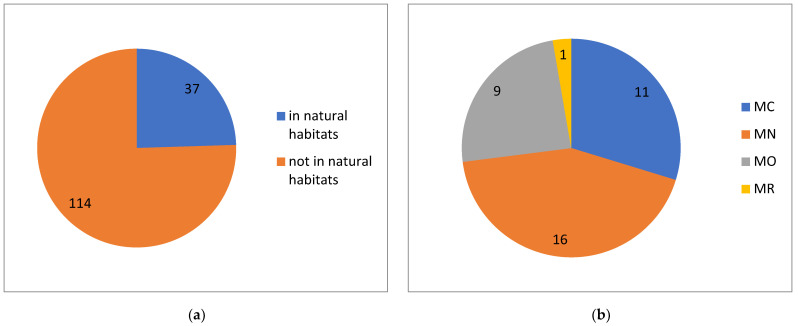
Occurrence of alien plants of Kyrgyzstan in natural habitats (**a**) with their estimated impact on ecosystems (**b**). Estimated impact [69]: Minimal Concern (MC), Minor (MN), Moderate (MO), Major (MR).

Although we have no exact data on the occurrence of alien plants and their particular habitat types, we know that only a quarter of their total number was encountered in nature, whereas the others are restricted to human-made habitats (urbanised and developed areas) (Figure 9a).

Only one species is estimated to have major negative impact on natural ecosystems, and nine species are considered to have moderate negative impact (Figure 9b). These 10 species are tentatively classified as invasive (Table 1). In this category, the members of Asteraceae (six species) clearly dominate, followed by Convolvulaceae (two species). More than one species are included in the genera *Cuscuta*, *Erigeron* and *Xanthium* (two species each).

Among the alien plants found in natural ecosystems, 28 species are considered to have a minor impact or to be of minimal concern (Figure 9b).

**Figure 10:**
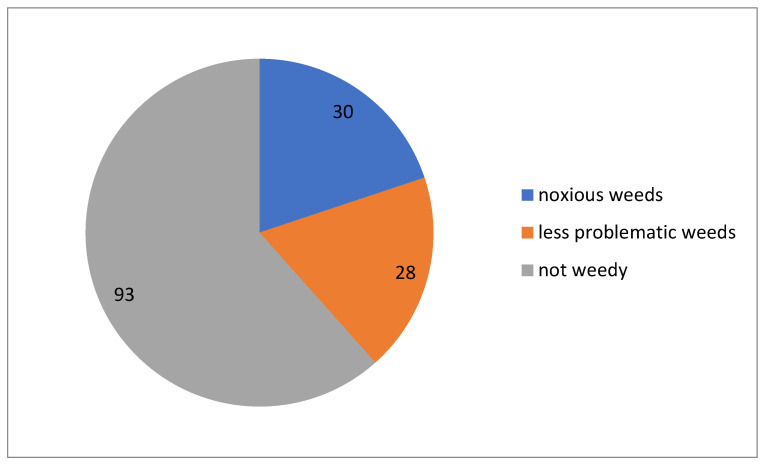
Proportion of weedy plant species with their estimated impact among alien plants in Kyrgyzstan.

Less than 40% of alien plants in Kyrgyzstan are weedy (Figure 10), i.e., found exclusively or significantly occurring in cultivated areas. About half of these can be considered noxious weeds.

**Figure 11:**
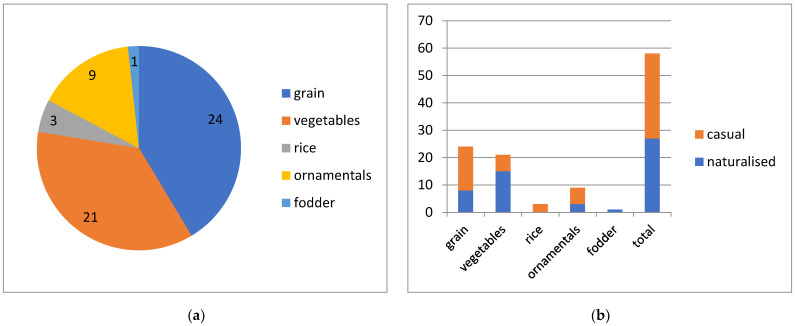
Prevalence of weedy alien plants of Kyrgyzstan in certain types of crops (**a**) with their success of naturalisation (**b**).

We classified the alien weeds according to the type of crops infested by these plants (Figure 11a). The greatest number of alien species was found in grain crops and vegetable gardens (40% each). Ca. 15% were encountered as weeds of ornamental plants, whereas only three species were specific weeds of rice. A single species (*Cerastium nemorale*) was found only in fodder crops.

Among weedy alien plants, the greatest proportion of naturalised species (70%) was found in vegetable gardens and technical crops. About one third of the weeds are naturalised grain crops and ornamental plants, but none were rice weeds (Figure 11b). Altogether, nearly half of the total number of weeds are naturalised at least locally.

**Figure 12:**
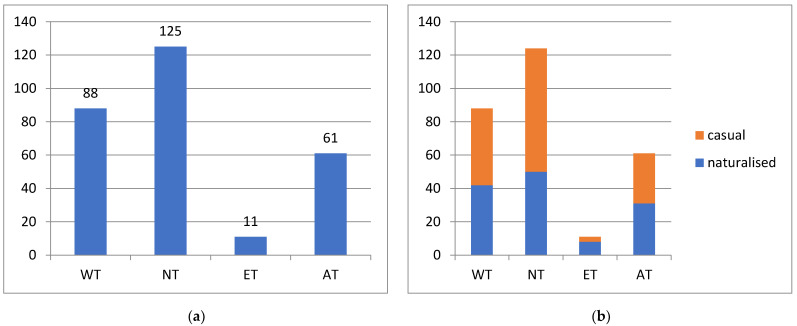
Distribution of alien plants in phytogeographic regions of Kyrgyzstan (**a**) with their success of naturalisation (**b**). For the names and locations of the regions, please see Figure 1.

The alien plant species are unevenly distributed in Kyrgyzstan (Figure 12a). The highest number of species (83% of the total) was registered in Northern Tian-Shan, followed by Western Tian-Shan (59%) and the Alay Mountain System (40%). Only 8% of the total diversity of alien plants was registered in Eastern Tian-Shan.

The proportion of naturalised plants is the lowest (40%) in Northern Tian-Shan, nearly 50% in Western Tian-Shan and Alay Mountain System, but exceeds 70% in Eastern Tian-Shan (Figure 12b).

## 4. Discussion

Our present verified count, i.e., 151 alien vascular plant species currently reported in Kyrgyzstan, nearly twice exceeds the counts of alien plants provided in 2014 by Lazkov & Sultanova [31], who considered that only 81 alien plant species were found as unintentionally introduced or outside their places of original cultivation. This increase can be partly explained by several new records published recently [50,51,53,54,59,60], but largely comes from a rigorous distinction between native species on one side and archaeophytes and old neophytes on the other side. This distinction, made for the first time in the present work, takes into account global distribution areas and species status in the neighbouring territories and Central Asia as a whole. Since we applied a modern classification and criteria to approach plant introductions in Kyrgyzstan [73] and took into account all published floristic research, our work can be considered the first comprehensive inventory of alien plants in the country that can be used in standardised global analyses of plant invasions.

As the total number of vascular plants in Kyrgyzstan is currently estimated at 3950 species, the level of synanthropisation of its flora is ca. 4%. This proportion is much lower in comparison to the European data, e.g., over 40% in the Czech Republic [74], although it stays at the same level as the proportion of alien plants in other arid Asian countries, e.g., 3.7% in Iran [72]. This low proportion of alien plants also agrees with the common situation in West Asia, which was explained by a lower level of botanical research in that region [75].

Since our recent revisions [53,54] highlighted deficiencies and gaps in the published floristic information, we expect that the current number of alien plants is far from the definite figure. Besides the actively on-going synanthropisation of the global floras [76], resulting in the flow of new additions, further changes may affect the status of some of the plant species listed in [31]. For example, we previously [54] re-assessed the status of *Physalis angulata*, which was listed in [31] as native but appeared to be encountered only in cultivation. Similarly, *Morus alba* and *M. nigra* were recorded as native in [31], whereas in the present work, we re-assessed their status, changing them to cultivated and casual alien (*M. alba*) and cultivated only (*M. nigra*).

Similarly, in our work, we changed the status of some species previously considered as common and presumably naturalised to casual because their current populations appeared not to be self-sustaining. The most notable example of such cases is *Xanthium strumarium*, which was treated as a common weedy and ruderal plant in [42], as less common in [45,46] and as nearly extinct in [53].

The task of separating old, naturalised plants from true native flora is a real challenge. For example, some native plant species (e.g., *Arctium lappa*) may appear on ruderal habitats due to their dispersal by humans and domestic animals. Plant species of arid foothills (e.g., *Centaurea benedicta*) easily occupy disturbed lands and anthropogenic habitats and may casually appear as weeds, especially on fallow fields. Some species may have different residence status in different countries across Central Asia; e.g., *Arctium tomentosum* is native in Kazakhstan and northern Kyrgyzstan due to a more humid climate in the lower mountain belt, whereas it is a recent alien in southern Kyrgyzstan, Uzbekistan and Tajikistan [77,78] where the climate is more arid. Due to such difficulties, the residence status of old alien plants (archaeophytes) may be accepted as native in global checklists and databases, e.g., POWO [79]. The latter resource is currently under construction in regard to its distributional part, and due to the shortage of national assessments, not all occurrences in this database are complete or correctly evaluated yet (for example, *Salvia aethiopis* and *Senecio vulgaris* are accepted as native to Kyrgyzstan in this resource, whereas the former is a recent, actively spreading neophyte [48,80], and the latter is a rare, casual garden weed introduced from Russia).

The apparent dominance of casual aliens over naturalised plants can be explained by the harsh climate of Kyrgyzstan [53], whose high aridity and continentality together with predominantly high elevations do not allow for the naturalisation of tropical species or those from the boreal or hemiboreal zone of Eurasia [81]. Annual (or monocarpic) life cycle may be considered an adaptation to the arid climate [82], thus explaining a higher proportion of annual or monocarpic alien plants in Kyrgyzstan. However, the level of naturalisation of alien plants in Kyrgyzstan (40%) is pretty high and is at the maximal limit found in temperate Asia [18]. As the total number of aliens is not high, the lower proportion of casual aliens seems to be another result of the harsh climatic conditions, which may suppress the appearance of many potential casual aliens.

The apparent success of Mediterranean plants in plant invasions of Kyrgyzstan can be explained by the close climatic conditions of the Mediterranean and the Central Asia because the same hot-summer Mediterranean climate [83] can be found in and around Uzbekistan, the closest neighbour of Kyrgyzstan. The relatively low number of alien plants originated from the New World (20%) stands at odds with the recent observations in Iran, where the greatest proportion of the total alien species and their naturalised part originated from that continent, presumably due to the wider diversity of Iranian ecosystems [72]. This observation suggests that climatic control indeed plays a key role in plant introduction and naturalisation in Central Asia.

Plant families with the leading number of alien species are Asteraceae, Brassicaceae and Poaceae, as shown in many other studies [72,84], but the contribution of the first two families is by far the greatest. Asteraceae are considered among the most successful plant families in the world, especially in arid and mountainous areas [85], and their highest presence among alien plants in Kyrgyzstan makes no surprise. Similarly, Brassicaceae and Poaceae along with Fabaceae and Amaranthaceae also keep their leading positions in the native flora of Kyrgyzstan [31]. Due to their lesser representation, the position of other families in the chart seems to be stochastic.

So far, we have collected no data on the times of introduction (first records), but the periods of introduction [53,54] can be analysed. We consider 13% of alien plants in Kyrgyzstan to have arrived in the Neolithic era, or at least before the Arabic rule was established in Central Asia in the 8th century. Notable examples of such plants are old crops (e.g., *Alkekengi officinarum* [54]) and weeds (e.g., *Solanum nigrum*, *Solanum villosum* and *Xanthium strumarium* [53]). A comparable number of species seemingly arrived with ancient cultivation during the period of Muslim states in Central Asia (e.g., *Hemerocallis fulva* [54]). However, by far the greatest number of alien plants (65%) were introduced after the Russian conquest of Central Asia, since the mid-19th century, when the territory was part of the Russian Empire and then part of the Soviet Union. This figure reflects the remarkable improvement of agriculture and subsequent industrialisation of the country, which caused a flood of alien plants arriving with intentional introduction, as weeds or contaminants, or as stowaway plants in ore mining and processing (e.g., [48]). The most recent period of independent Kyrgyzstan, beginning in 1991, brought 14% of the new alien plants, but its short duration suggests that the intensity of plant invasions during this period should be at least at the same level as in the Soviet times.

The number of alien species with high impacts on natural ecosystems, which can be considered invasive, is about 6.5%, which is similar to nearly 4% found in Iran [72], but comprises a quarter of all alien plant species found in natural habitats in Kyrgyzstan. Some of these species (e.g., *Ailanthus altissimus*, *Erigeron annuus* and *Pilosella aurantiaca*) have limited distributions in the country but show the features of possible aggressive expansion in the future. Whereas the naturalisation of *Ailanthus altissimus* and *Erigeron annuus* is a recent phenomenon, *Pilosella aurantiaca* was already naturalised in the Tian-Shan Mountains in the 1950s, to the extent that when its secondary distribution area was encountered by local botanists, it was erroneously described as a presumed national endemic [42]. *Erigeron canadensis* and *Xanthium spinosum*, other alien species with rather limited distributions in the country, can be regularly found in native landscapes around many populated places. Two other American plants that are widely distributed elsewhere, e.g., in Europe [80], are recent neophytes in Kyrgyzstan: *Bidens frondosa* and *Matricaria discoidea*. These species have been recorded in a few places but cannot be considered invasive yet due to the limited size of their populations [54].

Asteraceae are the leading family among alien species with higher negative impact on nature. Although plants from the New World constitute only 20% of the total number of plant aliens in Kyrgyzstan, they comprise a half of the most harmful plant species in nature. This observation agrees with a high number of American aliens in Iran, which absolutely dominate the national blacklist [72].

Pathways of introduction, although not recorded at this stage of research, can be partly inferred from the usage of alien plants in Kyrgyzstan. Slightly over a third of all alien plant species were cultivated at some stage in their history (although not necessarily nowadays). This cultivation may indicate their major pathway of introduction. The highest proportion of ornamental plants reflects their increasing role in recent plant introductions and invasions [75], although the level of naturalisation of such plants in Kyrgyzstan is very low. The greatest proportion of naturalised medicinal plants reflects their ancient, now abandoned use in Central Asia [54]; among such old introductions, only self-sustained populations survived (e.g., *Hyoscyamus niger*), whereas *Matricaria chamomilla*, the only casual immigrant in this category, is a recent introduction. Edible plants demonstrated zero success in naturalisation, having been maintained by humans despite their negligible adaptivity to the local climate.

The other 40% of alien plants in Kyrgyzstan are weedy and introduced most likely as contaminants. Their high level of naturalisation (nearly 50%) suggests that weeds are a major contributor to the synanthropic flora in the country. Other alien species (ca. 30%), which are neither weedy nor cultivated, arrived as contaminants of various commodities or as stowaway (neither precisely treated nor distinguished as separate pathways yet).

The major parts of weedy plants are associated with grain fields and vegetable gardens, the traditional agricultural components in Central Asia. The role of weeds of ornamental plants (including those in nurseries and greenhouses) is much less significant but most likely underestimated due to the lack of dedicated research. Rice weeds, which played a greater role in Uzbekistan [77,86,87,88,89,90], occupy a negligible place because of the too minor proportion of tropical cultures in Kyrgyzstan [45,46]. The greatest level of naturalisation can be observed in weeds associated with vegetables, followed by those associated with grain fields. Grain crops played a major role in the national economy together with the cultivation of vegetables and technical plants (like tobacco and cotton), especially in the Soviet times, when most of the aliens arrived.

The distribution of alien plant species according to phytogeographic regions of Kyrgyzstan [53] is highly uneven. As agriculture is concentrated mostly in the warm lowland depressions and only present to a minor extent in the higher mountain valleys [91], and the largest cities with the highest level of import and development are situated in the same area, this concentration explains the high number of alien plant species along the northern (WT) and southern (AT) sides of the Fergana Depression and in the Chüy Depression (NT). The strikingly low diversity of alien plants in the inner Tian-Shan (ET) could be determined by its harsh climatic conditions: the prevalence of elevations over 2000 m and an extreme continentality.

Future studies on the alien plants of Kyrgyzstan, as exemplified by our recent contributions [53,54], should determine more precise time, pathways, routes and vectors of their introduction. Although native distribution areas are important characteristics of alien plants, many of them may arrive to the territory through a different continent, from secondary distribution areas. Such studies reveal a rich background of plant invasions and uncover important trends in their processes and patterns of their establishment [92].

In general, the alien flora of Kyrgyzstan requires thorough field exploration and monitoring, for which currently no botanical resources exist. We believe that our inventory provides a solid foundation for future studies, for which a national programme for alien plant invasion control should be established as soon as possible.

## 5. Conclusions

The harsh climatic conditions of Kyrgyzstan (high aridity and continentality) determine the low total number of alien plant species and the low proportion of casual aliens in this number. The highest number of alien plant species in Kyrgyzstan originated from the Mediterranean, which can be explained by some common climatic features between this area and Central Asia. Half of ten most harmful aliens originated from the Americas, showing a different trend. Whereas the legacy of the early agricultural period and the early Muslim states of Central Asia still remains in a considerable number of alien plant species, the intensity of plant invasions was by far the greatest during the period of the Russian Empire and the USSR; this rapid accumulation of alien plants continues in independent Kyrgyzstan. The uneven distribution of alien plants in the territory of Kyrgyzstan is explained by the different elevations and continentality of its regions, as well as by the concentration of agricultural activities and human population along warm lowland depressions.

## Figures and Tables

**Table 1:** Invasive plant species of Kyrgyzstan, their life cycle, origin and estimated impact.

Family	Scientific Name	Life Cycle	Period	Native Area	Occurrence	Impact
Asteraceae	*Erigeron annuus*	monocarpic perennial	independence	America	local	moderate
Asteraceae	*Erigeron canadensis*	annual	Russian	America	wide	moderate
Asteraceae	*Pilosella aurantiaca*	perennial	Russian	C Europe	local	moderate
Asteraceae	*Xanthium orientale*	annual	Russian	America	regional	moderate
Asteraceae	*Xanthium spinosum*	annual	Russian	America	regional	moderate
Asteraceae	*Xeranthemum annuum*	annual	Russian	Mediterranean	regional	moderate
Convolvulaceae	*Cuscuta campestris*	annual	Russian	America	regional	major
Convolvulaceae	*Cuscuta chinensis*	annual	Russian	China	regional	moderate
Orobanchaceae	*Phelipanche aegyptiaca*	annual	pre-historic	Mediterranean	wide	moderate
Simaroubaceae	*Ailanthus altissima*	perennial	Russian	China	local	moderate

## Data Availability

The data presented in this study are available as Supplementary Materials to this article and as a taxonomic checklist with occurrence data published via Global Biodiversity Information Facility: https://doi.org/10.15468/jymaru.

## Supplementary Materials

The following supporting information can be downloaded at: https://www.mdpi.com/article/10.3390/plants13020286/s1. Table S1: annotated checklist of alien plants in Kyrgyzstan.
